# Molecular Karyotyping of Human Single Sperm by Array- Comparative Genomic Hybridization

**DOI:** 10.1371/journal.pone.0060922

**Published:** 2013-04-02

**Authors:** Cristina Patassini, Andrea Garolla, Alberto Bottacin, Massimo Menegazzo, Elena Speltra, Carlo Foresta, Alberto Ferlin

**Affiliations:** Department of Molecular Medicine, Section of Clinical Pathology and Centre for Human Reproduction Pathology, University of Padova, Padova, Italy; IGBMC/ICS, France

## Abstract

No valid method is currently available to analyze the entire genome of sperm, including aneuploidies and structural chromosomal alterations. Here we describe the optimization and application of array-Comparative Genomic Hybridization (aCGH) on single human sperm. The aCGH procedure involves screening of the entire chromosome complement by DNA microarray allowing having a molecular karyotype, and it is currently used in research and in diagnostic clinical practice (prenatal diagnosis, pre-implantation genetic diagnosis), but it has never been applied on sperm. DNA from single human sperm isolated by micromanipulator was extracted, decondensed and amplified by whole-genome amplification (WGA) and then labeled, hybridized to BAC array, and scanned by microarray scanner. Application of this protocol to 129 single sperm from normozoospermic donors identified 7.8% of sperm with different genetic anomalies, including aneuploidies and gains and losses in different chromosomes (unbalanced sperm). On the contrary, of 130 single sperm from men affected by Hodgkin lymphoma at the end of three months of chemotherapy cycles 23.8% were unbalanced. Validation of the method also included analysis of 43 sperm from a man with a balanced translocation [46,XY,t(2;12)(p11.2;q24.31)], which showed gains and losses corresponding to the regions involved in the translocation in 18.6% of sperm and alterations in other chromosomes in 16.3% of sperm. Future application of this method might give important information on the biology and pathophysiology of spermatogenesis and sperm chromosome aberrations in normal subjects and in patients at higher risk of producing unbalanced sperm, such as infertile men, carriers of karyotype anomalies, men with advanced age, subjects treated with chemotherapy, and partners of couples with repeated miscarriage and repeated failure during assisted reproduction techniques.

## Introduction

Chromosomal aneuploidies (trisomy or monosomy) and structural aberrations are found in about 1% of newborn, 6% of still live births, 8% of clinically recognized pregnancies, 50% of spontaneous abortions, and 50–60% of embryos and blastocysts generated *in vitro* during assisted reproduction techniques (ART) [1], [2]. Therefore, not only they are responsible for a large number of miscarriages, represent the leading cause of pregnancy loss and the leading genetic cause of developmental disabilities and mental retardation [1], but also they are important limiting factors for the success of natural conception and *in vitro* fertilization techniques, which in general are characterized by low implantation and high abortion rates. At birth, the incidence of aneuploidy (0.33%) and structural abnormalities (0.25%) is similar [3]. While the maternal effect is predominant for some of such anomalies, many aneuploidies (such as those for the sex chromosomes) and most structural chromosomal rearrangements are paternal in origin [1], [3]–[5]. Although some data are available for sperm aneuploidies, the existing information on *de novo* rearrangements (such as duplications and deletions) in human sperm is very limited [4], [5]. The high number of cellular divisions occurring during spermatogenesis favors the origin of unbalanced sperm, in particular as a consequence of meiotic errors at the spermatocyte level [1], [3]–[5]. The errors cannot be eliminated because of the absence of repair mechanisms in the final steps of spermiogenesis, the transition from spermatids to mature sperm [1], [3]–[5]. Categories of subjects at risk of producing higher percentage of unbalanced sperm are represented by carriers of reciprocal and robertsonian translocations [6], infertile men, carriers of Yq microdeletions [7], subjects with Klinefelter syndrome [8], men with advanced age [9], subjects treated with chemo-and radiotherapy, partners of couples with repeated miscarriage and repeated ART failure [10], and fathers of Klinefelter, Down and Turner subjects.

To date, studies aimed at identifying chromosomal anomalies in sperm have been performed by sperm karyotyping after fusion of spermatozoa with hamster eggs or by fluorescent *in situ* hybridization (FISH) with two or more DNA probes on decondensed sperm nucleus [4], [5]. Both techniques have however several technical and applicative problems, so the knowledge on the physiology and pathophysiology of the origin of chromosomal alterations in human sperm (especially structural aberrations) are still not well understood. The analysis of sperm karyotyping using chromosome banding techniques is the only method to simultaneously detect numerical and structural abnormalities of any chromosome in human sperm, but it is very laborious and it can analyze a limited number of spermatozoa. Therefore, most recent studies used multicolour FISH, which however allows the screening of aneuploidy of a limited number of chromosomes, or the analysis of structural abnormalities only of specific chromosomes. Furthermore, it is prone to hybridization failure and it cannot detect simultaneously numerical and structural alterations of all chromosomes.

Therefore, an alternative method to study chromosomal sperm alterations is needed. We reasoned that a comprehensive chromosomal screening technique such as array Comparative Genomic Hybridization (aCGH) could be a valid tool to this aim.

The aCGH procedure involves screening of the entire chromosome complement by DNA microarray allowing having a “molecular karyotype” at higher resolution that traditional karyotype and allowing overcoming diagnostic limits of FISH. The development and clinical application of aCGH in last years has revolutioned the diagnostic process in several diseases and aCGH is gradually joining or replacing standard cytogenetic techniques in a growing number of genetic and molecular laboratories [11]–[15]. It allows the identification of aneuploidies and unbalanced chromosome alterations, even submicroscopic (less than 3–10 Mb, not determined by standard karyotype analysis), and it is currently used in research and in diagnostic clinical practice (prenatal diagnosis, pre-implantation genetic diagnosis). Array CGH has never been applied to sperm.

Here we present the development and optimization of aCGH on single human sperm. The developed protocol includes isolation and aspiration of single sperm by micromanipulator, lysis of sperm, DNA extraction, sperm DNA decondensation, whole-genome amplification (WGA), aCGH, hybridization to BAC array, and scanning by microarray scanner.

## Subjects, Materials and Methods

The study was approved by the Ethics Committee of Azienda Ospedaliera Padova (protocol 2401P) and each participant provided written informed consent.

### Subjects

Three volunteer men of proven fertility provided semen samples and, after confirming they were normozoospermic (sperm concentration >15 mill/mL, total sperm count >39 mill/ejaculate, forward motility >32%, normal morphology >4%), 50 single sperm from each subject were selected. Similarly, 50 single sperm were studied from a man with a balanced translocation 46,XY,t(2;12)(p11.2;q24.31), and from three patients affected by Hodgkin lymphoma at the end of three months of chemotherapy cycles (ABVD, adriamycin-doxorubicin, bleomycin, vinblastine, dacarbazine).

**Figure 1:**
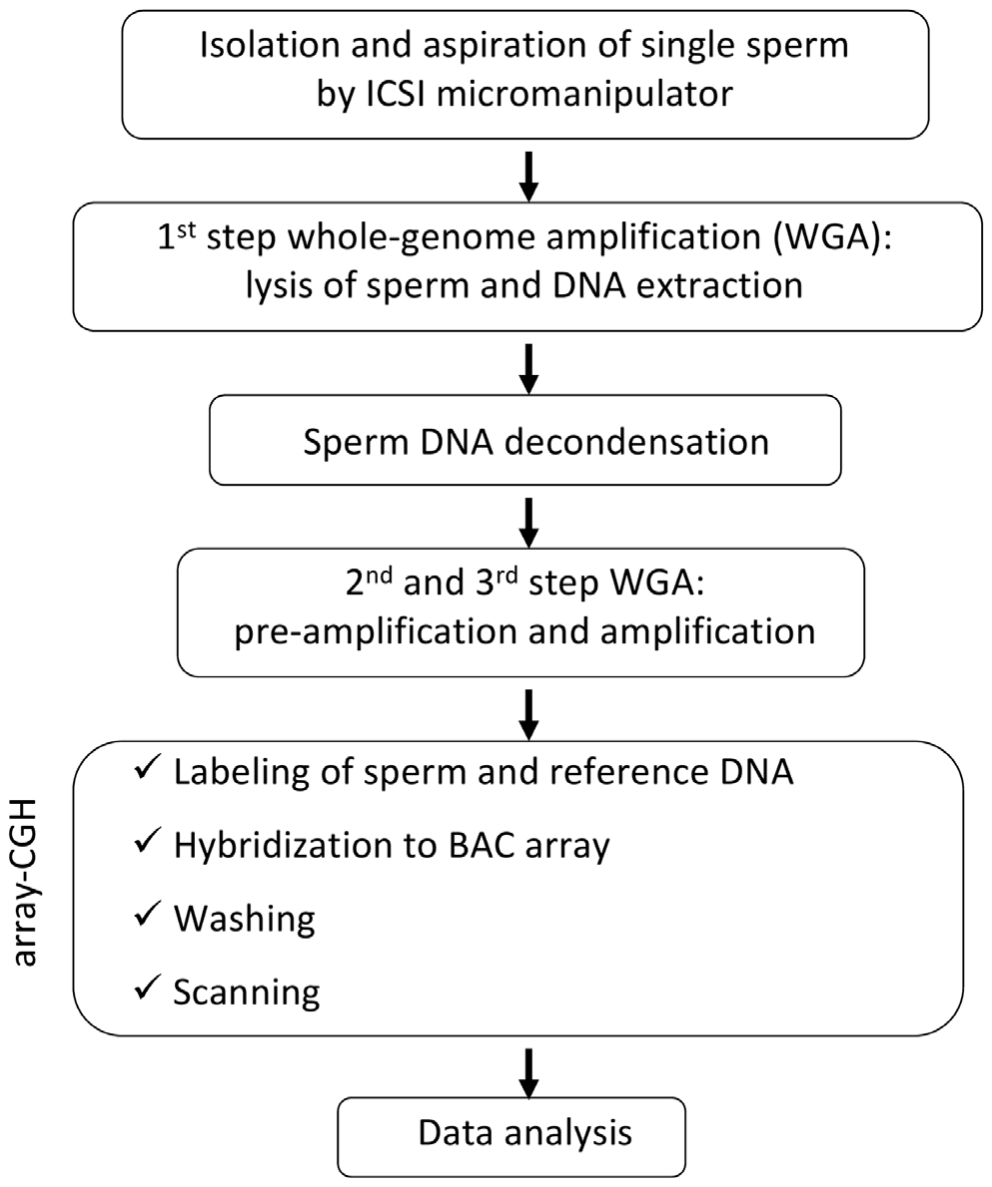
Flow diagram of the protocol developed for aCGH on single human sperm.

The whole protocol described below is summarized in figure 1.

### Isolation and aspiration of single sperm

Semen samples were first analyzed following WHO recommendations [16], then washed twice by centrifugation at 300 g for 10 min in sterile phosphate-buffered saline (PBS) and a small aliquot was placed on Petri dish and processed with a micromanipulator used for ICSI (intracytoplasmic sperm injection) (Nikon Eclipse TE2000-U) equipped with Nomarski optics, an HMC MC2 ×20/0.40 objective lens. Individual motile sperm with normal morphology were then aspirated and each placed into a 0.2 ml PCR tube with 2 µl of PBS

### Lysis and extraction of sperm DNA and sperm decondensation

The SurePlex DNA Amplification System (SurePlex WGA, BlueGnome, Cambridge, UK) was used to extract and amplify the DNA from the single sperm. Amplification is highly representative with low allele drop out which makes the resulting product ideal for use with BlueGnome's 24sure aneuploidy screening arrays. The protocol is formed by a lysis phase and two rounds of PCR amplification for WGA. SurePlex uses a WGA procedure based on 3 steps: extraction and random fragmentation of genomic DNA, pre-amplification reaction and subsequent amplification reaction by PCR utilizing flanking universal priming sites. In the first step the samples were centrifuged at 1800 g for 3 min. at 4°C and 3 µl of cell extraction buffer were added to each sample. The extraction and fragmentation reaction contained 4.8 µl of extraction enzyme dilution buffer and 0.2 µl of cell extraction enzyme for each sperm (total volume of 10 µl), which were incubated in PCR thermocycler at 75°C for 10 min. and at 95°C for 4 min. In the second step 5 µl of SurePlex Pre-Amplification mix were added to each 10 µl of sample. SurePlex Pre-Amplification mix contained 4.8 µl of SurePlex Pre-Amp buffer and 0.2 µl of SurePlex Pre-Amp enzyme. The samples were incubated according to the following thermalcycler program: one cycle at 95°C for 2 min. followed by 12 cycles at 95°C for 15 s, 15°C for 50 s, 25°C for 40 s, 35°C for 30 s, 65°C for 40 s and 75°C for 40 s. In the third step 60 µl of Amplification mix were added to the 15 µl synthesis reaction product. The Amplification mix contained 25 µl of SurePlex Amplification buffer, 0.8 µl of SurePlex Amplification enzyme and 34.2 µl of water. The synthesis reaction products were cycled according to the following PCR conditions: 95°C for 2 min. followed by 14 cycles at 95°C for 15 s, 65°C for 1 min. and 75°C for 1 min. To determine the success of amplification, 5 µl of each amplified sample were loaded on a 1% agarose 1× TAE gel.

The decondensation of sperm DNA was performed between the first and the second step. The best condition of decondensation was obtained by incubating the single sperm with proteinase K (700 nM) at 56°C for 10 min. and then with DTT (1 mM) at 37°C for 30 min.

### Whole genome amplification (WGA)

Once the decondensation protocol has been optimized, the WGA procedure has been adapted in order to improve the sensitivity and the accuracy of the amplification. For this purpose the last PCR-based cycling step in SurePlex has been developed in Real-Time PCR, adding SYBR Green at the final concentration 0.125×. This Amplification mix contained 25 µl of SurePlex Amplification buffer, 0.8 µl of SurePlex Amplification enzyme, 4.68 µl of Sybr Green (2×) and 29.52 µl of water. The synthesis reaction products were cycled according to the following Real-Time PCR conditions: 95°C for 2 min. (data collection for baseline reading) followed by 14 cycles at 95°C for 15 s, 65°C for 1 min. and 75°C for 1 min. (data collection). Data analysis was performed by subtracting the baseline for fluorescence levels at the end of each cycle, and the difference in fluorescence was plotted against the cycle number.

### Array-CGH

Then we proceeded to array-CGH, which is divided into four steps: labeling, combination and centrifugal evaporation, hybridization and washing.

In the first step, equal amount of sperm DNA and reference DNA (SureRef. Reference male DNA) were differentially labeled with Cyanine 3 (Cy3) e Cyanine 5 (Cy5). The two labeling reaction mixes contained: 5 µl of reaction buffer, 5 µl of primer solution, 5 µl of dCTP-labelling mix and 1 µl of Cy3 dCTP or Cy5 dCTP respectively. 16 µl of Cy3 labeling mix were added to 8 µl of amplified sperm DNA and 16 µl of Cy5 labeling mix were added to 8 µl of SureRef reference DNA (total volume of 24 µl). These samples were denatured in a thermalcycler for 5 min. at 94°C and then transferred immediately to ice for 5 min. Then, 1 µl of Klenow enzyme was added to each PCR tube to proceed with the labeling reaction in a thermalcycler for 2–4 h at 37°C.

In the second step, labeled sperm DNA and control DNA were combined, then co-precipitated adding 25 µL of COT Human DNA and concentrating each sample by a centrifugal evaporator for 1 at 60°C, until around 3 µl remained in each tube.

In the third step, labeled DNA was resuspended in 21 µl prewarmed dextran sulphate hybridization buffer, denatured at 75°C for 10 min. and hybridized under cover slips to 24sure BAC-arrays (BlueGnome). Then 24sure slides were placed in prepared hybridization chamber (slide box, tissue saturated with 6 ml 2× SSC/50% formamide) and incubated in a non-circulating lidded water bath for 16–20 h at 47°C.

In the last step, hybridized 24sure slides were washed to remove un-hybridized DNA. The cover slip was removed from each slide by manually agitating in 2× SSC/0.05% Tween 20 at room temperature. Afterwards each slide was washed according these conditions: first wash (on stirrer) in buffer 2× SSC/0.05% Tween 20 at room temperature for 10 min.; second wash (on stirrer) in buffer 1× SSC at room temperature for 10 min.; third wash in buffer 0.1× SSC at 60°C for 5 min.; fourth wash (on stirrer) in buffer 0.1× SSC at room temperature for 1 min. Finally, each slide was dried by centrifugation at 170 g for 6 min.

A laser scanner (G2565CA microarray scanner, Agilent, USA) was used to read the resulting images of the hybridization. The result of the co-hybridization is the emission of two distinct signals (red for Cy5 and green for Cy3) whose ratio indicates whether the amount of DNA is equivalent or whether there is a gain or loss at specific chromosomal regions. Scanned images were then analyzed and quantified by BlueFuse Multi Software (BlueGnome). The difference in fluorescence between sample DNA and reference DNA was plotted against all chromosomes and signals above or below the fluorescence thresholds indicate respectively gains and losses of the DNA content.

Once the array-CGH 24sure protocol has been optimized, this procedure of analysis of single sperm has been further modified in order to improve sensitivity and reproducibility, using 24sure V3 protocol in which test and reference data come from different sub arrays. A reference sub array consists of SureRef Male DNA run against Sureref Female DNA, labeled with contrasting dyes, on a single sub array: SureRef Male DNA labelled with Cy3 against Sureref Female DNA labelled with Cy5 and SureRef Male DNA labelled with Cy5 against Sureref Female DNA labelled with Cy3. The differences with respect to 24sure protocol were: in a PCR tube 5 µl of primer solution were combined with 8 µl of amplified sperm DNA or SureRef reference DNA. These samples were denatured in a thermalcycler for 5 min. at 94°C, then transferred immediately to ice for 5 min. 12 µl of Cy3 or Cy5 labeling master mix were added to the 13 µl of the sample DNA/primer solution and SureRef DNA/primer solution to proceed with the labeling reaction in a thermalcycler for 2–4 h at 37°C. The two labeling reaction mixes contained: 5 µl of reaction buffer, 5 µl of dCTP-labeling mix, 1 µl of Cy3 dCTP or Cy5 dCTP respectively and 1 µl of Klenow enzyme. The other steps remained unchanged.

## Results and Discussion

We developed for the first time a protocol of aCGH to obtain a “molecular karyotype” on single human sperm. This method allows the analysis of the entire genome and identifies aneuploidies and chromosomal alterations with high resolution and accuracy. The developed protocol (Fig. 1) includes: i) isolation and aspiration of single sperm by a micromanipulator used for the selection and injection of single sperm into oocytes for ART (intracytoplasmic sperm injection – ICSI); ii) lysis of sperm, DNA extraction, sperm DNA decondensation and whole-genome amplification (WGA); iii) aCGH (labeling of sperm DNA and reference DNA with Cyanine 3 (Cy3) and Cyanine 5 (Cy5), hybridization to BAC array, washing, and scanning by microarray scanner).

The most critical steps in the development of the protocol were the optimization of an efficient method of chromatin decondensation (sperm chromatin is highly condensed thanks to the substitution of histones with protamines) that allows to and does not interfere with subsequent WGA and DNA hybridization for aCGH, and the adaptation of aCGH protocol to one single haploid cell. Different conditions of decondensation have been tested using several combinations and concentrations of NaOH, SSC, DTT, EDTA, SDS, and proteinase K at different temperatures and incubation times, starting from NaOH 1N and SSC 2× at room temperature that represents the current method used in our laboratory for sperm decondensation for aneuploidy analysis by FISH (Table S1). Aggressive protocols, (NaOH/SSC), yielded failure of hybridization of DNA of the sperm to the array due to the degradation of DNA and consequent non-amplification of the entire genome. The best condition has been obtained incubating single sperm with proteinase K (700 nM) at 56°C for 10 minutes and then with DTT (1 mM) at 37°C for 30 minutes.

**Figure 2:**
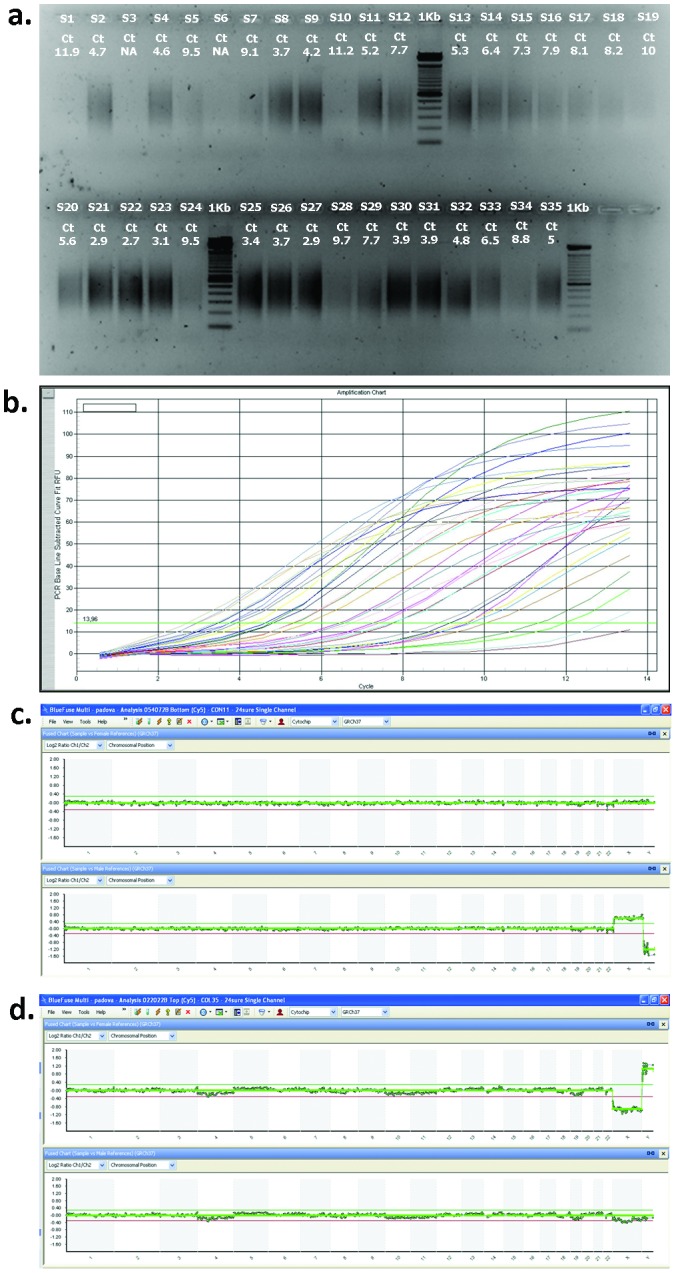
WGA analysis and aCGH of single sperm. **a**. Gel electrophoresis of WGA products from single human sperm. Ct: cycle threshold as determined by Real-Time PCR with SYBR Green. **b**. Real-Time PCR of the same WGA products. The difference in fluorescence (fluorescence at the end of each cycle minus baseline fluorescence level) is plotted against the cycle number. Hybridization failed for samples with a cycle threshold >10. **c**. Molecular karyotype of a normal 23,X sperm co-hybridized with female (upper panel) and male reference (lower panel). **d**. Molecular karyotype of a normal 23,Y sperm co-hybridized with a female (upper panel) and a male reference (lower panel).

In the standard protocol of WGA (SurePlex WGA, BlueGnome, Cambridge, UK) the analysis of the amplification products is performed by staining with SYBR safe DNA gel stain after electrophoresis on 1% agarose gel. The smear intensity on the gel depends on the efficiency of the decondensation and/or of WGA (Fig. 2a), and samples showing no smear or a weak smear underwent subsequent hybridization failure. We modified the WGA procedure to improve the sensitivity and the accuracy of the amplification. For this purpose the last PCR-based cycling step was performed in Real-Time PCR with SYBR Green. Data analysis was performed by subtracting the baseline for fluorescence levels at the end of each cycle, and the difference in fluorescence was plotted against the cycle number (Fig. 2b). This analysis enabled us to define a threshold of 10 cycles above which subsequent hybridization fails. Using this protocol, 85% of single sperm analyzed showed optimal amplification and hybridization.

**Figure 3:**
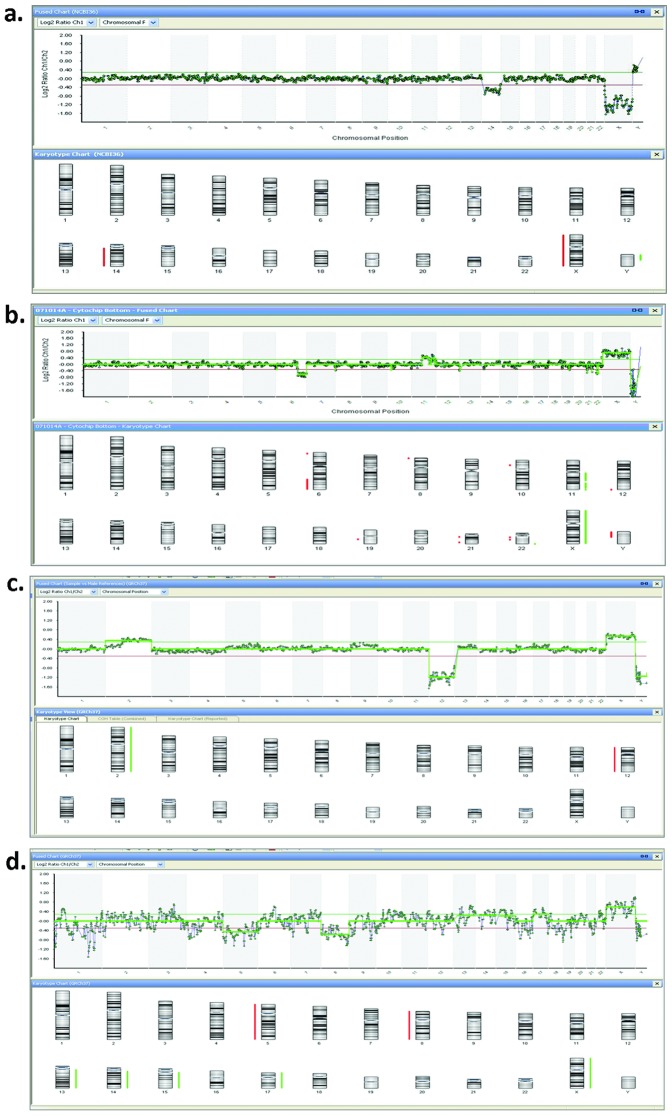
aCGH of abnormal single sperm. **a**. Molecular karyotype of a sperm with a loss of the entire chromosome 14 (arr 14p13q32.33(1–106,864,802)×0) from a man with normozoospermia. **b**. Molecular karyotype of a sperm with a loss of part of chromosome 6 and a gain of part of chromosome 11 (arr 6q22.31q27(123,028,998–170,784,959)×0; arr 11q12.1q25(59,009,184–134,070,242)×2) from a man with normozoospermia. **c**. Molecular karyotype of an unbalanced sperm from a men with a balanced translocation t(2;12)(p11.2;q24.31): arr 2p11.2q37.3(86,951,334–242,565,564)×2 and arr 12p13.33q24.33(1–133,851,895)×0. **d**. Complex molecular karyotype of a sperm from a patient after chemotherapy.

For aCGH, equal amounts of sperm DNA and reference DNA (control) are labeled with Cy3 and Cy5 using random primers, the labeling mixes are then combined, co-precipitated, concentrated by centrifugal evaporator, resuspended and then hybridized for 16–20 hours at 47°C under cover slips to 24sure BAC-arrays (BlueGnome), a BAC array covering the entire genome (autosomes and sex chromosomes) with a resolution of 10 Mb. Hybridized 24sure slides are then washed to remove unbound labeled DNA, and a laser scanner (G2565CA microarray scanner, Agilent, USA) is used to read the resulting images of the hybridization. The result of the co-hybridization is the emission of two distinct signals (red for Cy5 and green for Cy3) whose ratio indicates whether the amount of DNA is equivalent or whether there is a gain or a loss at specific chromosomal regions. Scanned images are then analyzed and quantified by BlueFuse Multi Software (BlueGnome). The difference in fluorescence between sample DNA and reference DNA is plotted against all chromosomes and signals above or below the fluorescence thresholds indicate respectively gains and losses of the DNA content (Fig. 2 and 3). We further optimized the procedure in order to improve sensitivity and reproducibility, using 24sure V3 protocol in which test and reference data come from different sub arrays including male and female DNA references labeled with contrasting dyes, allowing sex match and sex mismatched analysis and hence clearer interpretation of sex chromosome aneuploidies (Fig. 2c and 2d).

After optimization of the entire procedure, in each single sperm experiment more than 95% of the clones was hybridized with success, the signal of the background noise was very low, the hybridization value was 1500–2000, the percentage of clones included was >90%, the Mean Spot Amplitude Ch1/Ch2 was >1200 (hybridization index), and the SBR Ch1/Ch2 was >4 (index of the background noise).

The optimized protocol was then applied to study the molecular karyotype of single sperm from normozoospermic men. Three men of proven fertility provided semen samples and, after confirming they were normozoospermic, 50 single sperm from each subject were selected. Successful decondensation, WGA and aCGH were obtained in 129 sperm, which showed a normal karyotype in 119 cases (92.2%). Ten sperm (7.8%) showed different anomalies, including aneuploidies of the sex chromosomes (2 XY disomy) and gains and losses in different chromosomes (loss of the entire chromosome 14, Fig. 3a; partial loss of ch. 6 and partial gain of ch. 11, Fig 3b; partial gain of ch. 12; partial loss of ch. 4, 5 and 12; partial loss of ch. 6; partial loss of chromosome 16; loss of the entire ch. 17; partial loss of ch. 20, 21, 22).

To further test the validity of the method in identifying abnormal karyotypes, 50 single sperm from a man with a balanced translocation 46,XY,t(2;12)(p11.2;q24.31) were analyzed. Eight sperm (8/43 that correctly amplified, 18.6%) showed gains and losses corresponding to the regions involved in the translocation (unbalanced sperm) and 7 (7/43, 16.3%) showed alterations in other chromosomes (partial loss of ch. 3; partial gain of ch. 7, 9, 14; 2 partial gain of ch. 13; partial gain of ch. 14; partial gain of ch. 16 and 17; partial gain of ch. 18) (interchromosomal effect). Finally, we analyzed 150 sperm from three men at high risk of producing unbalanced sperm (patients affected by Hodgkin lymphoma at the end of three months of chemotherapy cycles). This analysis showed that 31/130 amplified sperm (23.8%) showed abnormal karyotype, including sex chromosome aneuploidies (4 XY disomy, 3 XX disomy, 1 sex ch. nullisomy), gains and losses in different regions of different chromosomes (n: 17), or complex alterations (n: 6). Examples of representative sperm are shown in figure 3, and a summary of the results is presented in table 1.

**Table 1:** Subject characteristics and summary of aCGH results on single sperm.

Subjects characteristics	Age (years)	Single sperm aCGH results	
		N. of aberrant sperm	Type of aberration
Controls (fertile normozoospermic)	31, 33, 34	10/129 (7.8%)	XY disomy (x2); Loss of single entire chromosomes (x2); Loss and/or gain of part of chromosomes (x6)
Reciprocal translocation [46,XY,t(2;12)(p11.2;q24.31)]	34	15/43 (34.9%)	Gains and losses of the chromosomal regions involved in the translocation (unbalanced sperm) (x8); Loss and/or gain of part of chromosomes (x7)
Hodgkin lymphoma (end of three months of ABVD chemotherapy)	28, 30, 36	31/130 (23.8%)	XY disomy (x4); XX disomy (x3); Sex chromosome nullisomy (x1); Loss and/or gain of part of chromosomes (x17); Complex abnormal (x6)

In conclusion, we developed for the first time a protocol of aCGH to obtain a “molecular karyotype” on single human sperm. This method allows the analysis of the entire genome and identifies aneuploidies and chromosomal alterations with high resolution and accuracy. We chose to analyze single sperm with a BAC aCGH because this technique has already been tested and validated on single cells (blastomere and polar body), which obviously contains a very limited amount of DNA. Furthermore, although BAC arrays have a lower resolution than oligonucleotide arrays, they reduce the likelihood of detecting genetic alterations untrue. Finally, the BAC clone targets have been mapped to the human reference sequence produced by the International Human Genome Sequencing Consortium, allowing easy access to information in the related genomic databases. This technique is able to easily detect segmental amplification of whole chromosome arms, terminal deletions, and discrete, high magnitude copy number gains and losses of all chromosomes. However, as with all CGH platforms, the arrays cannot detect epigenetic changes, balanced translocations, mitochondrial DNA aberrations or point mutations.

Future application of this method might give important information on the biology and pathophysiology of spermatogenesis and sperm chromosome aberrations in normal healthy fertile subjects, as well as in patients at higher risk of producing unbalanced sperm, such as infertile men, carriers of karyotype anomalies, men with advanced age, subjects treated with chemotherapy, and partners of couples with repeated miscarriage and repeated ART failure. Although BAC aCGH is highly sensitive and in general allows detecting Copy Number Alterations (CNAs) in samples with a high percentage of contaminating normal cells, future studies are necessary to verify its accuracy in detecting chromosomal alterations in pooled semen samples. In fact, a part from conditions predisposing to producing a high number of unbalanced sperm, a relative high number of sperm should be analysed to detect abnormalities occurring at relatively low frequency (10 of 129 sperm in normozoospermic men in this study). This is particularly important considering the high cost of such analysis.

## Supporting Information

Table S1 **Conditions of sperm DNA decondensation tested.**
(DOC)
