# Supplementary material for: Molecular Karyotyping of Human Single Sperm by Array- Comparative Genomic Hybridization
Source: PLoS One. 2013 Apr 2;8(4):e60922. doi: 10.1371/journal.pone.0060922 (PMC3614952; doi:10.1371/journal.pone.0060922)
Supplement: Table S1 — Conditions of sperm DNA decondensation tested. (DOC) [file pone.0060922.s001.doc]

**Table S1.** Conditions of sperm DNA decondensation tested.

| NaOH 1N + SSC 2X RT (standard protocol for aneuploidy analysis by FISH) |
| --- |
| DTT 1 mM 37°C x 30’ |
| DTT 5 mM 37°C x 30’ |
| DTT 10 mM 37°C x 30’ |
| DTT 1 mM 37°C x 1h |
| SDS 1% + EDTA 5mM 37°C x 1h |
| DTT 40 mM + EDTA 5 mM 37°C x 1h |
| SDS 1% + DTT 10 μM 37°C x 15’ |
| proteinase K 700 nM 56°C x 10' |
| proteinase K 700 nM 56°C x 10' + DTT 1 mM 37°C x 30' |
